# Suicide-related features in migrant people with a recent suicide attempt: Results from the SURVIVE Study

**DOI:** 10.1192/j.eurpsy.2025.280

**Published:** 2025-08-26

**Authors:** A. De La Torre-Luque, M. Diaz-Marsa, Y. Sanchez-Carro, L. J. Gonzalez-Agudelo, M. Elices, M. Botí, A. Cebria, P. Saiz, M. Ruiz-Veguilla, P. Lopez-Peña, A. Gonzalez-Pinto, A. Palao, V. Perez-Sola

**Affiliations:** 1 Complutense University of Madrid; 2 CIBERSAM ISCIII; 3 Hospital Clinico San Carlos, Madrid, Spain; 4 La Concordia University, Montreal, Canada; 5 Parc Sanitari Hospital Mar; 6 Clinic Hospital, Barcelona; 7 Parc Tauli, Sabadell; 8 University of Oviedo, Oviedo; 9 Hospital Virgen del Rocio, Sevilla; 10 Hospital Araba Vitoria, Vitoria-Gasteiz; 11 La Paz University Hospital, Madrid, Spain

## Abstract

**Introduction:**

Migrant people may constitute a vulnerable population with an increased risk of suicide-related behaviour due to the accumulation of multiple risk factors, such as migration-related stress, the history of traumatic experiences and socioeconomic situation in the country of immigration.

**Objectives:**

To study the prevalence of suicide attempts from migrant population in hospital emergency departments. Moreover, it aimed to study suicide-related outcomes, according to migration status.

**Methods:**

Data from 754 patients (73.1% female; m= 40.23, sd= 15.72) with a recent suicide attempt from 10 Spanish hospitals were included. Assessment protocols were delivered within the 15 days after the index attempt. Suicide-related outcomes, clinical and sociodemographic factors were assessed by administering a wide range of clinical tools (C-SSRS, MINI, BIS-21, BSI, ACSS-FAD, CTQ).

**Results:**

One in four patients was foreign-born, mostly being from Latin American countries (74% of foreign-born patients). Foreign-born patients were younger, higher psychopathology symptom severity, child trauma scores (Figure 1), than their counterparts (p < .01). Higher proportion of employed people and lower amount of people receiving pension benefits, were found in the foreign-born group. No between-group differences were observed regarding suicide-related outcomes. Finally index attempt in foreign-born group was featured by using more lethal methods (p < .05) (Figure 2).

**Image 1:**

**Figure:**
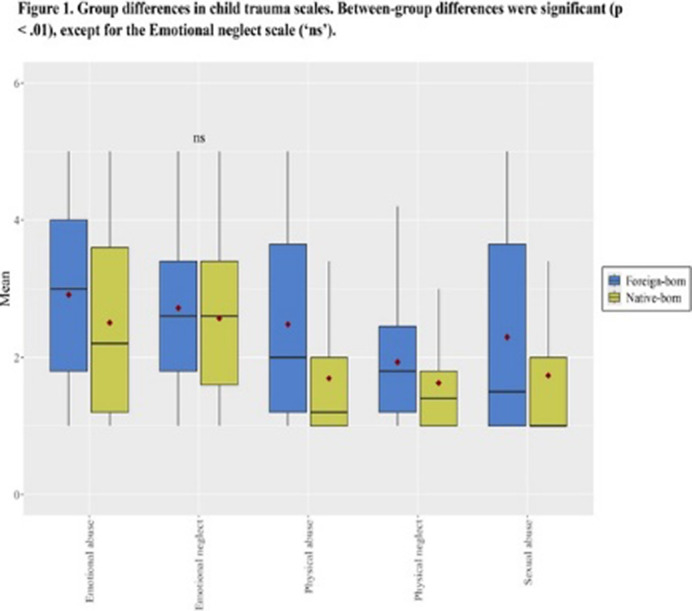

**Image 2:**

**Figure:**
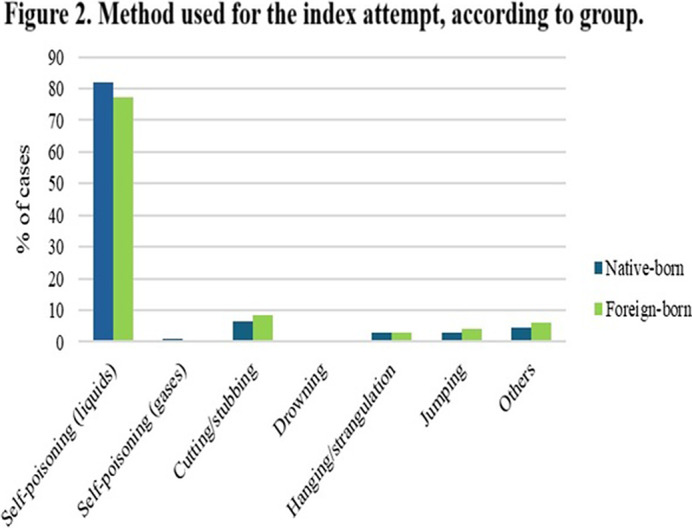

**Conclusions:**

Significant proportion of attempts attended in clinical settings may come from migrant people, mainly featured by child trauma history. Attempts from migrant populations may be featured by more lethal methods. Health care provision adjustment becomes mandatory to meet migrant people needs in current times.

**Disclosure of Interest:**

None Declared

